# Prevalence and assessment tools of cancer-related cognitive impairment in lung cancer survivors: a systematic review and proportional meta-analysis

**DOI:** 10.1007/s00520-024-08402-9

**Published:** 2024-03-05

**Authors:** Mu-Hsing Ho, Tsz Wei So, Chun Lun Fan, Yiu Tak Chung, Chia-Chin Lin

**Affiliations:** 1 School of Nursing, LKS Faculty of Medicine, The University of Hong Kong, 5/F, 3 Sassoon Road, Academic Building, Pokfulam, Hong Kong SAR; 2 Faculty of Medicine, The Chinese University of Hong Kong, Shatin, Hong Kong SAR; 3 School of Life Science, Faculty of Science, The Chinese University of Hong Kong, Shatin, Hong Kong SAR; 4 Faculty of Science, The University of Hong Kong, Pokfulam, Hong Kong SAR

**Keywords:** Cancer nursing, Cancer-related cognitive impairment, Meta-analysis, Prevalence

## Abstract

**Purpose:**

Cancer-related cognitive impairment (CRCI) is a significant risk factor influencing the quality of life in lung cancer survivors. No absolute assessment tool has been confirmed to assess CRCI in lung cancer survivors. This review was undertaken to pool the overall prevalence of CRCI and to summarize the assessment tools in assessing CRCI among lung cancer survivors.

**Methods:**

PubMed, Cochrane Library, Embase, CINAHL, and CNKI were searched to retrieve articles reported CRCI prevalence. Summary prevalence estimates were pooled using a random effects model, along with corresponding 95% prediction intervals (PIs). The Freeman-Tukey double arcsine transformation of proportions was incorporated in the analysis. Additionally, subgroup analysis, meta-regression, and leave-one-out analysis were performed.

**Results:**

A total of 12 studies, involving 1934 survivors, were included in the review. All of these studies were found to have a low risk of bias in terms of their methodological quality. Four studies (33.3%) utilized the International Cognition and Cancer Task Force (ICCTF) criteria to identify CRCI through neuropsychological tests. The pooled prevalence rate of CRCI was found to be 26% (95% PI, 16–37%), *I*^*2*^ = 95.97%. The region in which the studies were conducted was identified as a significant factor contributing to this heterogeneity (*p* = 0.013). No indication of small-study effects was found (Egger’s test: *p* = 0.9191).

**Conclusion:**

This review provides an overview of CRCI prevalence and assessment tools in lung cancer survivors. The findings can serve as epidemiological evidence to enhance clinicians’ and researchers’ understanding of early detection and assessment.

**Supplementary Information:**

The online version contains supplementary material available at 10.1007/s00520-024-08402-9.

## Introduction

Lung cancer is highly prevalent and stands as the primary cause of cancer-related deaths on a global scale [1]. Surgery (i.e., lung resection) remains the most efficient treatment among lung cancer survivors in the early stage [2]. For advanced stage or those who are unable to undergo surgery, their primary option for treatment is likely to be chemotherapy and/or other combined treatments [3]. Cancer-related symptoms have been well-studied and found to be the risk factors influencing the quality of life and overall survival in lung cancer survivors. However, insufficient research on cognitive changes has created a significant gap in our knowledge, leaving cognitive impairment as a crucial missing piece of the puzzle that requires further investigation [4] and dysfunction among lung cancer survivors have been undertaken in regard to the mechanism, prevalence, and its management.

Cancer-related cognitive impairment (CRCI) is defined as cognitive dysfunction experienced by individuals who have survived cancer, resulting in impairments in areas such as working or short-term memory, attention, executive functions, orientation, language comprehension, and processing speed [5]. In cancer research, there is a growing emphasis on examining the effects of cognitive functioning on the adverse outcomes of cancer and its treatments. The cognitive function of cancer survivors can be influenced not only by the progression of the disease itself but also by the treatments administered to combat it [6]. Around 15–25% of survivors with breast cancer present objective cognitive decline [5]. The International Cancer and Cognition Task Force (ICCTF) has found that the impact of different cancer treatments and underlying mechanisms on the development of CRCI is substantial [7]. CRCI has a significant impact on the functional independence and overall quality of life for individuals who are living with cancer. Additionally, it contributes to the development of physical and psychological issues, including anxiety, depression, fatigue, and sleep disturbances [5, 8]. It is also found that survivors are not fully aware of the cognitive functioning decline caused by the cancer treatments; clinical recognition and psychoeducation about CRCI are likely the most crucial aspect of its management. Often, cancer survivors, their families, and practitioners were just considered that the cognitive dysfunction was perhaps due to the decline in general health and it might be because of cancer, the disease itself [9, 10]; highlighting the clinical recognition, awareness, and health education for them is crucial.

Various risk factors have been identified for CRCI, including advanced age, lower levels of cognitive functioning prior to undergoing cancer treatments such as chemotherapy, surgery, radiation therapy, and combined therapy, cancer-related symptoms, and physical inactivity. [11–13]. A recent comprehensive review concluded that increased physical activity and exercise have a positive effect in reducing the occurrence of CRCI. However, the relationship between physical activity and CRCI in lung cancer survivors remains uncertain due to limitations in existing studies. These limitations include the lack of evidence on long-term effects and the absence of an adequate comparison group that specifically addresses the patterns of physical activity [13–16]. A review that summarized various interventions for CRCI concluded that a combination of cognitive stimulation and physical activity is the most effective supportive care for cancer survivors with CRCI in clinical settings [17]. Despite some intervention studies having been implemented yet, the optimal strategy and intervention is not found. Practical evidence such as prevalence and cognitive assessment tools for identifying CRCI is remaining limited [18]. In particular, there is a lack of high-quality evidence investigating CRCI among lung cancer survivors. As the incidence of lung cancer continues to rise, the number of cancer survivors who are struggling with neurotoxicity has also increased significantly. Therefore, evidence regarding the prevalence data in lung cancer survivors is needed to underpin the clinical recognition and awareness of CRCI. To address the literature gaps, this systematic review and proportional meta-analysis aimed to pool the prevalence estimates of CRCI in lung cancer survivors.

For the CRCI assessment, increasing research have been undertaken using subjective measure to assess perceived cognitive impairment in cancer populations. For example, the European Organization for the Research and Treatment of Cancer Quality of Life Questionnaire (EORTC QLQ-C30) cognitive functioning subscale was commonly adopted. However, the major flaw is that the particular domains of cognitive function such as verbal learning, executive functions, language comprehension, and orientation are unable to be assessed using EORTC QLQ-C30 cognitive functioning subscale. However, it does not conclude that self-reported instruments are not encouraged to be used. There are several well-designed self-reported cognitive assessment tools, including Functional Assessment of Cancer Therapy-Cognitive Function (FACT-Cog) or PROMIS Cognitive Abilities and Cognitive Concerns Scales, which are recommended by the Cancer Neuroscience Initiative Working Group for assessing CRCI [19].

Cognitive function is complex and worth further investigating among lung cancer survivors in both objective and subjective observations. There is still ongoing debate regarding the relationship between objective and subjective cognitive problems, with complaints often being associated with psychological factors [5]. In sum, there is no absolute assessment tool that has been confirmed to assess CRCI and no evidence on whether self-reported measures are not comparable to objective assessments such as neuropsychological tests using the ICCTF criteria. The significance of this meta-analysis is that the overall prevalence and subgroup prevalences of objective or subjective types of measure, as well as the cognitive assessment tools used, are reported. The aim of this review is to pool the overall prevalence of CRCI and to summarize the assessment tools in assessing CRCI among lung cancer survivors.

## Methods

### Design

This systematic review and proportional meta-analysis adhered to the reporting guidelines of the Preferred Reporting Items for Systematic Reviews and Meta-Analyses (PRISMA) [20] and Meta-analysis of Observational Studies in Epidemiology (MOOSE) [21] reporting guidelines to ensure accurate and transparent reporting of the findings. The review protocol was registered in PROSPERO ID: CRD42023403279.

### Search methods

The search for relevant studies encompassed five electronic databases, namely PubMed, Cochrane Library, Embase, CINAHL, and CNKI, from their inception until May 2023. The search terms were formulated based on the core components of MeSH (Medical Subject Headings) to create an effective search strategy. The keywords utilized pertained to the condition under investigation, the context of the study, and the specific population or patient group of interest [22]: “Cognitive Dysfunction” [MeSH] AND “Cancer-Related Cognitive Impairment” [text] AND “Lung Neoplasms” [MeSH]. To tailor the search strategy to the specific requirements of each database, the combination of keywords was adjusted accordingly (Table S2).

### Inclusion and exclusion criteria

The inclusion criteria for articles in this review were as follows: (1) publication in a peer-reviewed journal in the English language, (2) reporting of cases related to cancer-related cognitive impairment, and (3) focusing on lung cancer survivors. This review included prospective, retrospective, and cross-sectional designs. Four authors (MHH, TWS, CLF, and YTC) screened and evaluated the titles and abstracts of all records independently. Review articles, case reports, conference papers, and letters to the editor that did not provide prevalence data were excluded from the study. Additionally, studies with low methodological quality were identified and excluded through a methodological quality assessment.

### Search outcome and data abstraction

To facilitate data abstraction, we developed a specific data collection sheet. Four authors (MHH, TWS, CLF, and YTC) independently retrieved the relevant data from the selected articles. The extracted information included details such as the authors’ names, publication year, region of the study conducted, research design, sample size, male ratio, age distribution, inclusion and exclusion criteria, the tool used for assessing CRCI, prevalence of CRCI, and identified risk factors for CRCI. A consensus was achieved through a comprehensive discussion among the four authors regarding the results of the data abstraction process.

### Quality appraisal

The Newcastle-Ottawa Scale (NOS) for scoring non-randomized trials in meta-analysis was used to evaluate the quality of the studies included in the analysis. For cohort studies, the maximum score for each study was 9 stars, and the NOS assessed three main aspects: (1) subject selection and exposure assessment (0–4 stars), (2) comparability between study groups (0–2 stars), and (3) adequacy of measurement and recording of study results and follow-up (0–3 stars). The total score provided an indication of the overall methodological quality of each study [23]. In the case of cross-sectional studies, the NOS assigns scores in three domains: selection (0–5 stars), comparability (0–2 stars), and outcome (0–3 stars). The total score for cross-sectional studies ranges from 0 to 10. Studies that achieve a total score above 7 are generally deemed to possess a high level of quality and exhibit a minimal risk of bias. This scoring system helps assess the methodological rigor and potential bias of cross-sectional studies included in the review [23, 24]. To ensure the accuracy and consistency of the quality assessment, each study was independently rated by two reviewers. In case of discrepancies in the results, a third investigator was consulted to reach a consensus. This approach helped minimize any potential biases and enhance the reliability of the evaluation process.

### Synthesis

All analyses were carried out using Stata SE version 18 statistics software (Stata Corp, College Station, TX). A random effects model was employed to pool the summary prevalence estimates, presented in percentages (%), along with their corresponding 95% prediction intervals (PIs) and confidence intervals (CIs). Additionally, the pooled proportions and weighted subgroups were also calculated. To visualize the pooled data, a graphical forest plot was created using Stata [25]. We used the Freeman-Tukey double arcsine transformation of proportions in the model and the *I*^*2*^ showed in the forest plot indicated the heterogeneity of the summary results. Given the inclusion of diverse populations and study locations, the researchers anticipated observing high levels of heterogeneity in their proportional meta-analysis. This heterogeneity can be attributed to contextual factors, such as the characteristics of the populations being studied and the geographical locations where the studies were conducted, which contribute to variations in the observed prevalence rates. As a result, it was expected that the meta-analysis would exhibit significant heterogeneity [26].

Subgroup analysis, meta-regression, and leave-one-out analysis were also performed. Subgroups based on regions and type of measures were compared. Meta-regression using the Stata meta-analysis package was conducted to explore potential sources of heterogeneity. Leave-one-out analysis was done to assess the influence of outliers. Egger’s test was used to assess small-study effects, and if necessary, the trim and fill method was used to correct for bias.

## Results

**Fig. 1:**
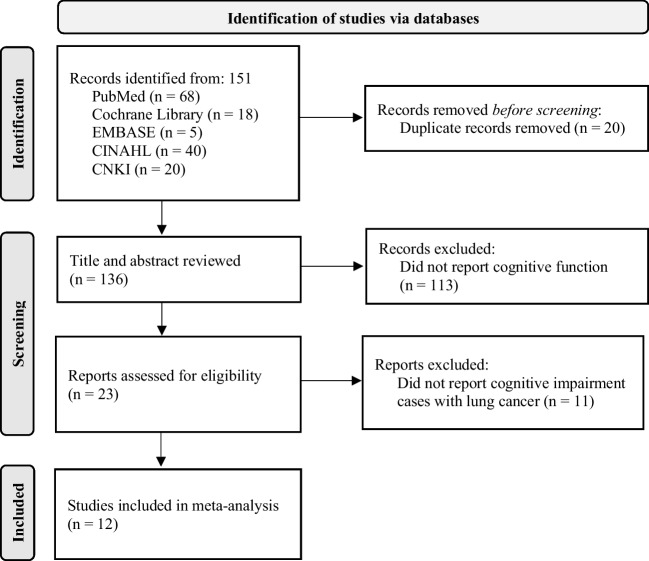
Flow diagram detailing the selection process of the included studies

The PRISMA flow diagram in Fig. 1 provides a visual representation of the study selection process for the current meta-analysis. Initially, a total of 151 published records were identified through searches in various databases. Specifically, 68 papers were found in PubMed, 18 papers in Cochrane Library, 5 papers in Embase, 40 papers in CINAHL, and 20 papers in CNKI. After removing duplicates, which amounted to 20 records, and excluding articles based on title and abstract screening, which accounted for 113 records, 23 full-text papers remained for further review. Among these, 12 studies involving a total of 1934 survivors were included in this meta-analysis. This selection process adheres to the PRISMA guidelines and ensures a systematic and transparent approach to study inclusion in the meta-analysis.

### Quality appraisal

All studies included in the meta-analysis were assessed for methodological quality and were found to have a low risk of bias. No studies were excluded based on suboptimal quality. The quality assessment, summarized in Table S1, was conducted by four reviewers to evaluate various domains of bias. The overall assessment indicates that the included studies are reliable and valid.

### Study characteristics

Table 1 lists the characteristics of the 12 studies published between 2008 and 2023. Most studies (*n* = 5; 41.6%) adopted a prospective study design [27–31]. In terms of the location and region where the studies were conducted, six studies were carried out in Asia [28, 29, 32–35], two studies in North America [27, 36], and four in Europe [30, 31, 37, 38]. The sample size of included studies ranged from 8 to 480; the ratio of male cancer survivors in the studies ranged from 46 to 100%. The mean age of survivors ranged from 59.1 to 68.8 years (Table 1).

**Table 1:** Characteristics of included studies (*n* = 12)

Author, year	Country, region	Design, sample size	Male (%)	Mean age	Inclusion criteria	Exclusion criteria	Assessment criteria	Risk factors
Bartels et al. (2021)	Berlin, Germany	Cross-sectional, 97	62.9	66^a^	Diagnosed of LC, regardless of subtype, stage, or treatment	Age ≥ 80 years, had brain metastases or a history of severe neurological or psychiatric disorders; received brain radiotherapy; had surgery less than 5 days before study enrolment	Neuropsychological tests by ICCTF criteria^b^ including VLMT, RAVLT, ROCF, the digit span forward and backward task, a component of the WMS, TAP, Stroop color-word test, Regensburger word fluency test, LPS, MWT-A, NART	Neuronal autoantibodies
Eggen et al. (2022)	Toronto, Canada	Cross-sectional, 77	51	62^a^	Age ≥ 18 years; diagnosed with mNSCLC	Primary brain tumors or psychiatric and neurological conditions	Neuropsychological tests by ICCTF criteria including HVLT-R TR, HVLT-R DR, TMTA, TMTB, COWA	Illness intrusiveness, self-esteem
Gal et al. (2020)	Multisite, Israel	Retrospective, 9	11.1	68.8	EGFR mutation or ALK rearrangement and symptomatic brain metastases that were treated with systemic therapy	NR	Medical record	NR
Grosshans et al. (2008)	Texas, USA	Prospective, 93	54	59^a^	Survivors who had previously experienced either a complete or partial response to concurrent chemoradiation, radiation alone, or surgical resection of their SCLC	NR	Neurocognitive Function Testing by ICCTF criteria^b^ including Verbal Selective Reminding Test and Benton Visual Retention Test, Wisconsin Card Sorting, number of perseverative errors, TMTB, WAIS-R, TMTA, COWA, grip strength, grooved pegboard test, and finger tapping	NR
Lu et al. (2023)	Shanghai, China	Prospective, 208	60.1	68.1	Aged ≥ 60 years; have been sent directly from the operating room to the ICU after surgery; diagnosis of LC confirmed by intraoperative pathology	Had a postoperative ICU stay <24 hours or >7 days	MMSE	Postoperative delirium
Luo et al. (2023)	Guangzhou, China	Cross-sectional, 378	68.5	59.1	Age ≥ 18 years; diagnosed with primary LC	Brain metastasis; history of neurological disease, psychiatric diseases, or psychotic episodes; other types of tumors	CogPCI	Diagnosed over 6 months, anxiety, leisure activity (protective factor), high platelet-to-lymphocyte ratio
Ma et al. (2023)	Multisite, China	Prospective, 480	64.8	60.8	Age ≥ 35 years; diagnosed with NSCLC; ECOG-PS < 5; treatment window period less than 3 months from prescription started.	Brain tumors or injury; history of stroke; active psychiatric diseases or narcotic usage, neurocognitive diseases; survivors who changed treatment protocols to chemotherapy; have taken antidepressant drugs	Neuropsychological tests by ICCTF criteria^b^ including TMT, HVLT-R, CANTAB battery (Cambridge Stockings to test spatial and planning memory)	NR
Schulkes et al. (2017)	Hague, The Netherlands	Prospective, 83	65	79^a^	Survivors with lung cancer aged 70 years and older referred for a geriatric assessment	NR	MMSE	NR
Shi et al. (2020)	Hohhot, China	Retrospective, 80	100	67.0	Age > 60 years; survivors with lung cancer who received VATL	Had distant metastasis, sublobar resection, or cognitive dysfunction before surgery; surgery could not be completed or received radiotherapy; had history of chest surgery, single lung ventilation, and extensive pleural adhesions	MMSE	NR
Soria-Comes et al. (2020)	Valencia, Spain	Retrospective, 70	85.7	77^a^	Survivors older than 70 years with a histopathological diagnosis of NSCLC	Diagnosed with an immunodeficiency, an autoimmune disease or a chronic infection (such as HIV or hepatitis)	SPMSQ or Pfeiffer test	Lower T-lymphocyte count (CD3+) (cells/mm^3^)
Takemura et al. (2022)	Hong Kong, China	Cross-sectional, 226	46	61.5	Diagnosed with stage IIIB, or IV NSCLC; ECOG-PS: 0–2; not engaged in regular exercise, defined as < 150 min of moderate-intensity exercise per week	Survivors diagnosed with neurological or psychiatric disorders	EORTC QLQ-C30 cognitive function subscale	Smoking, less daily step counts, fatigue, anxiety
Zeng et al. (2023)	Multisite, The Netherlands	Prospective, 134	64.5	60.8	Survivors with stage III NSCLC who did not show tumor progression after radical treatment	Survivors with central nervous system disease such as meningioma or psychiatric disorders, as these medical histories could influence the SRCF	EORTC QLQ-C30 cognitive function subscale	Baseline cognitive impairment

^a^median; ^b^according to the recommendations, of the International Cognition and Cancer Task Force (ICCTF) criteria, objective cognitive impairment was defined as two test score changes ≥1.5* SD from baseline scores or one test score ≥2* SD from baseline scores

*Abbreviations. LC* lung cancer, *ICCTF* International Cognition and Cancer Task Force, *mNSCLC* metastatic non-small cell lung cancer, *EGFR* epidermal growth factor receptor, *ALK* anaplastic lymphoma kinase, *ICU* intensive care unit, MMSE Mini-Mental State Examination, *CogPCI* Perceived Cognitive Impairments subscale, *ECOG-PS* Eastern Cooperative Oncology Group Performance Scores, *VATL* video-assisted thoracoscopic lobectomy, *SPMSQ* Short Portable Mental State Questionnaire, *EORTC QLQ-C30* European Organization for the Research and Treatment of Cancer Quality of Life Questionnaire, *NR* not reported, *VLMT* Verbal Learning and Memory Test, *RAVLT* Rey Auditory-Verbal Learning Test, *ROCF* Rey-Osterrieth Complex Figure test, *WMS* Wechsler Memory Scale, *TAP* Testbatterie für Aufmerksamkei, *LPS* Leistungsprüfungssystem, *MWT-A* Mehrfachwahl-Wortschatz-Intelligenztest-A, *NART* National Adult Reading Test, *HVLT-R TR* Hopkins Verbal Learning Test-Revised Total Recall, *HVLT-R DR* Hopkins Verbal Learning Test-Revised Delayed Recall, *TMT* Trail Making Test, *COWA* Controlled Oral Word Association, *WAIS-R* Wechsler Adult Intelligence Scale-Revised

### Identifying CRCI

Four studies (33.3%) reported that CRCI was assessed by neuropsychological tests using the International Cognition and Cancer Task Force (ICCTF) criteria [27, 29, 36, 37], three studies (25%) applied the Mini-Mental State Examination (MMSE) [28, 30, 34], and one study (8.3%) reported the application of the Short Portable Mental State Questionnaire (SPMSQ) [38]. Subjective assessments measuring perceived cognitive impairment for CRCI are frequently used as well. Three studies applied subjective assessments including Perceived Cognitive Impairments (CogPCI) subscale [33] and EORTC QLQ-C30 cognitive function subscale [31, 35]. Among neuro-psychological tests, a comprehensive list of cognitive domains was identified including verbal learning and memory, verbal fluency, expressive and receptive language, visuospatial memory, working memory, attention, executive function, processing speed and visual attention, mental flexibility, verbal and visual reasoning, and motor coordination. The corresponding measurements for these cognitive domains are summarized in Table 2.

**Table 2:** Summary of cognitive function assessment in lung cancer survivors

Assessment	Domain	Description
6. Objective		
Neuro-psychological tests/ Neurocognitive function tests	Verbal learning and memory	Verbal Learning and Memory Test^1^ HVLT-R^2, 6^ Verbal Selective Reminding Test^3^
	Verbal fluency, expressive and receptive language	COWA^2, 3^ Token Test^3^
	Visuospatial memory	Rey-Osterrieth Complex Figure test^1^ Benton Visual Retention Test^3^ CANTAB (Cambridge Stockings) ^6^
	Working memory	Digit span forward and backward^1,3^ Arithmetic subtests of the WAIS-R^3^
	Attention	Test of Attentional Performance^1^
	Executive function	TMT Part B^2, 3, 6^ Wisconsin Card Sorting^3^ Number of perseverative errors^3^
	Processing speed and visual attention	TMT Part A^2, 3, 6^ Digit Symbol subtest of the WAIS-R^3^
	Mental flexibility	TMT Part B^2,6^
	Verbal and visual reasoning	Similarities and Block Design subtests of the WAIS-R^3^
	Motor coordination	Grip strength^3^ Grooved pegboard test^3^ Finger tapping^3^
MMSE	Orientation	Orientation to time and place^4, 7, 8^
	Repetition	Repeating named prompts^4, 7, 8^
	Verbal recall	Repeating named prompts recall^4, 7, 8^
	Attention and calculation	Serial sevens^4, 7, 8^
	Language	Naming a pencil and a watch^4, 7, 8^
	Visual construction	Varies, involving drawing figure shown^4, 7, 8^
SPMSQ	Memory	Short-term and long-term memory^9^
	Orientation	Orientation to surroundings^9^
	Information	Information about current events^9^
	Serial mathematical tasks	Capacity to perform serial mathematical tasks^9^
7. Subjective		
CogPCI	Perceived cognitive impairment	Functional assessment of cancer therapy–cognitive scale^5^
FACT-Cog	Perceived cognitive impairment	Perceived cognitive impairment^6^ Perceived cognitive abilities^6^ Comments from others^6^ Impact on quality of life^6^
EORTC QLQ-C30 cognitive function subscale	Perceived cognitive function (memory and attention)	Have you had difficulty concentrating on things like reading a newspaper or watching television? (attention)^10, 11^ Have you had difficulty remembering things? (Memory)^10, 11^

*Abbreviations. COWA* Controlled Oral Word Association, *HVLT-R* Hopkins Verbal Learning Test-Revised, *CANTAB* Cambridge Neuropsychological Test Automated Battery, *WAIS-R* Wechsler Adult Intelligence Scale-Revised, *TMT* Trail Making Test, *MMSE* Mini-Mental State Examination, *CogPCI* Perceived Cognitive Impairments subscale, *SPMSQ* Short Portable Mental State Questionnaire, *FACT-Cog* Functional Assessment of Cancer Therapy, *EORTC QLQ-C30* European Organization for the Research and Treatment of Cancer Quality of Life Questionnaire

References in this table: 1[37]; 2[36]; 3[27]; 4[28]; 5[33]; 6[29]; 7[30]; 8[34]; 9[38]; 10[35]; 11[31]

### Pooled prevalence of CRCI

**Fig. 2:**
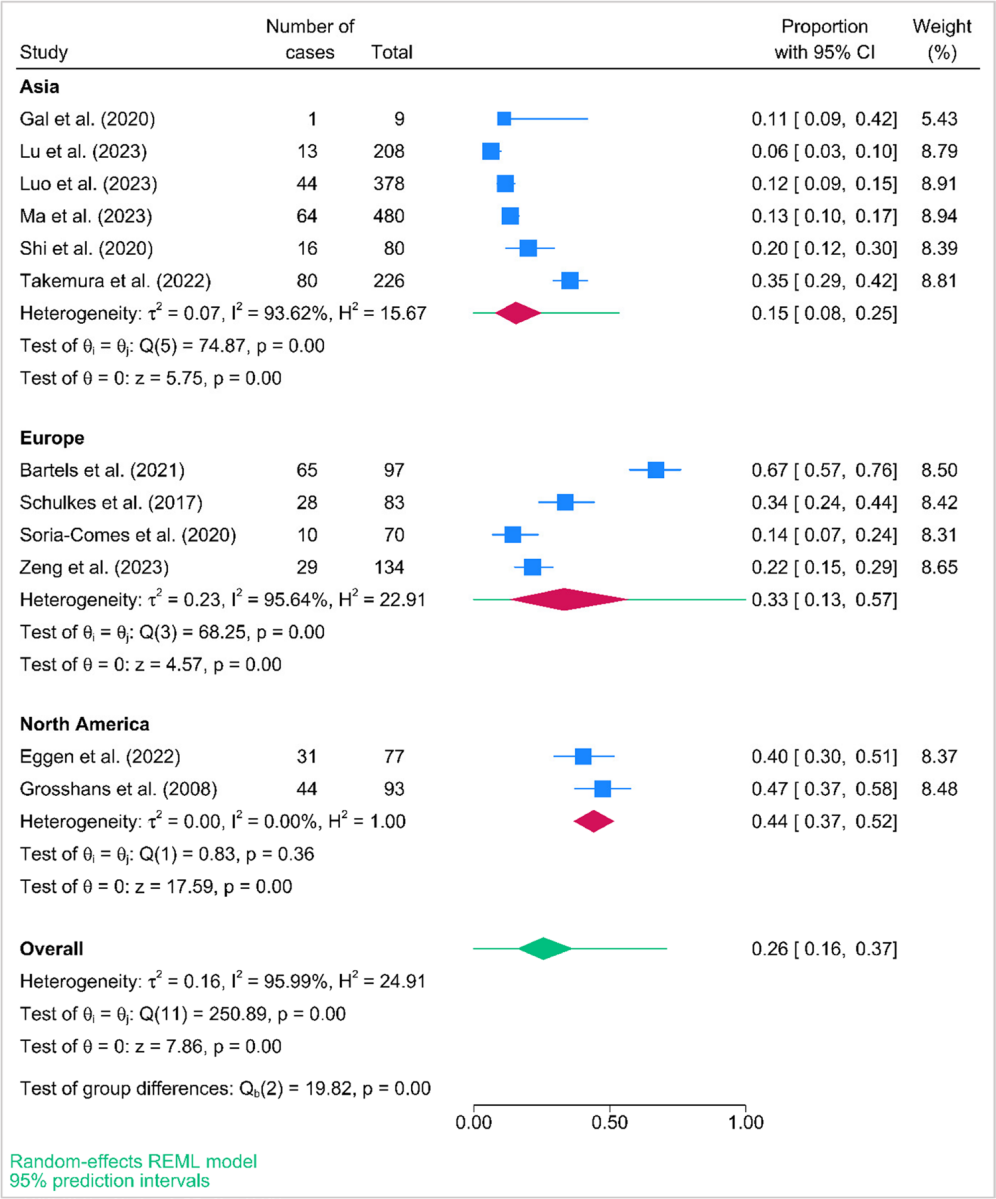
The forest plot of the overall pooled prevalence and study conducted in Asia, Europe, and North America reported estimates of cancer-related cognitive impairment from a random effects model

The prevalence of cancer-related cognitive impairment varied widely across the included studies, ranging from 6 to 84.4%. Figure 2 displays the forest plot, which presents the pooled prevalence estimates of the included studies. The summary prevalence rate of cancer-related cognitive impairment was observed to be 26% (95% PI, 16–37%). There was a high degree of heterogeneity between the studies, with an *I*^*2*^ value of 95.99%. This indicates substantial variability in the prevalence estimates among the included studies.

### Subgroup analysis and meta-regression

**Fig. 3:**
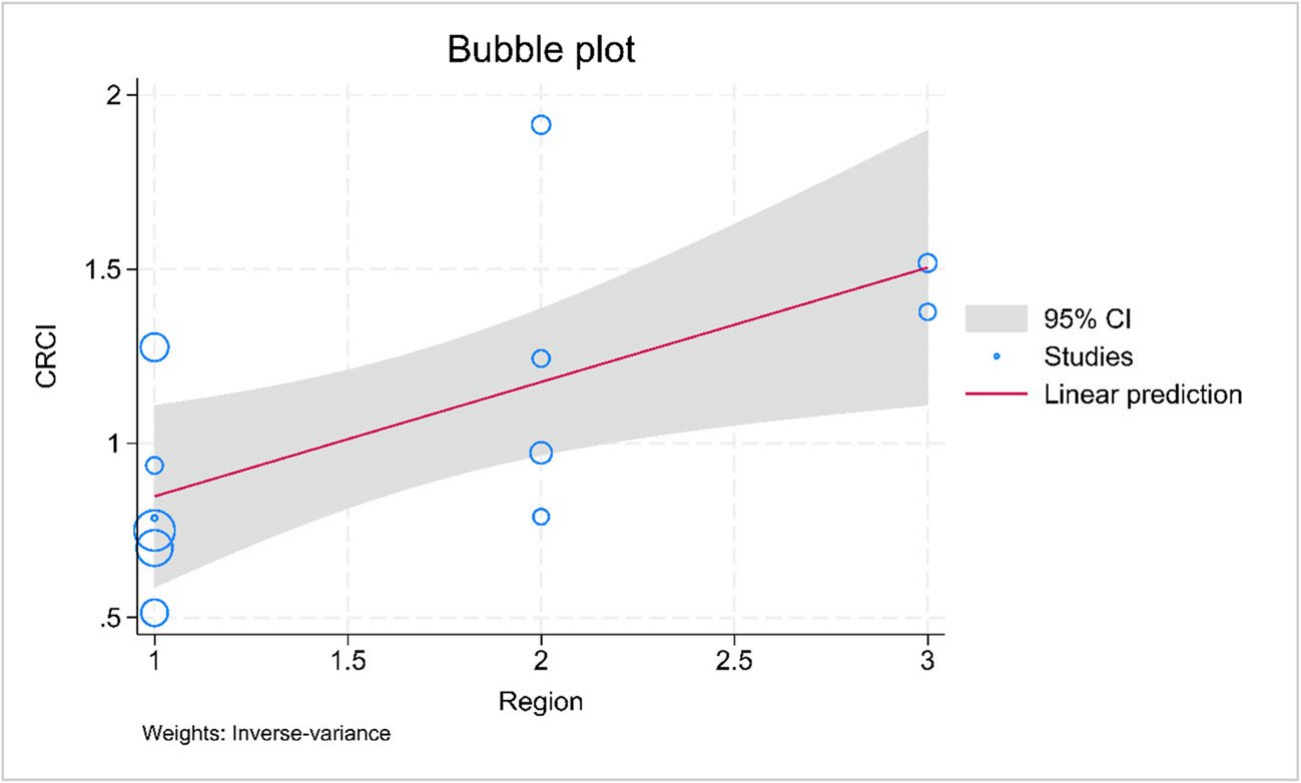
Bubble plot for region to display the result of meta-regression. *Note.* Region was found to be a significant factor contributing to heterogeneity (*p* = 0.017). CRCI, cancer-related cognitive impairment

Subgroup analyses were performed according to region (Asia/Europe/North America), and types of assessment (objective/subjective). The pooled prevalence estimates for Asia, Europe, and North America were 15% [95% PI, 8–25%], 33% [95% PI, 13–57%], and 44% [95% PI, 37–52%], respectively (Fig. 2). Our meta-regression analysis has shown that region (*p* = 0.013) was a factor associated with the heterogeneity. Figure 3 presents the bubble plot for the heterogeneity factor. As for types of assessment, the pooled prevalence estimates for objective measure using comprehensive evaluations was 36% [95% PI, 16–58%] and objective measure using screening tool alone was 17% [95% PI, 7–30%], while subjective measure was 22% [95% PI, 10–37%] (Figure S1). According to the meta-regression, the different types of assessment did not significantly contribute to the high heterogeneity observed.

### Leave-one-out analysis and small-study effects

**Fig. 4:**
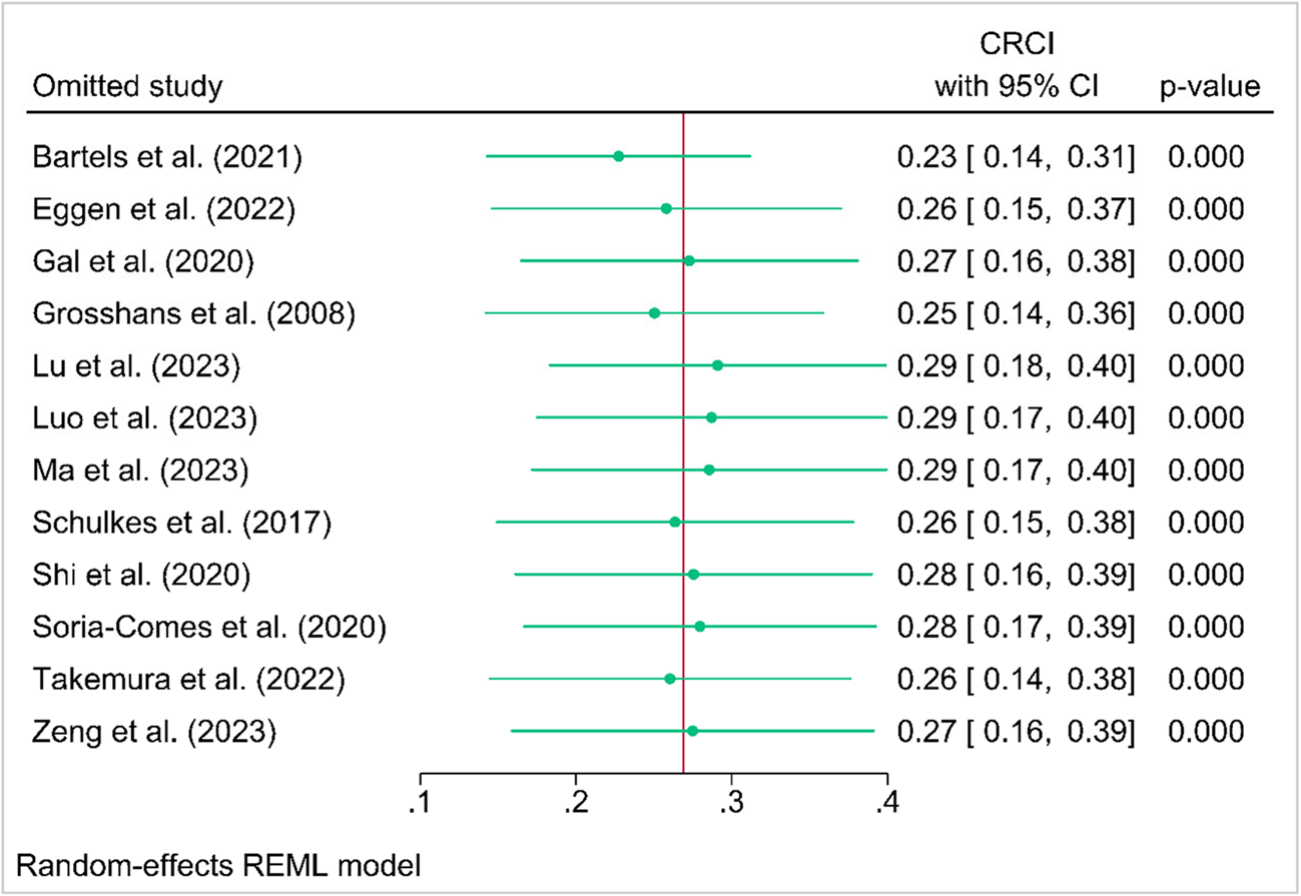
Leave-one-out analysis to identify the outlier and its impact. *Note*. Omitting study by Bartels et al. (2021) causes the overall prevalence of CRCI to decrease by 23% (overall prevalence of 12 studies was 26%). CRCI, cancer-related cognitive impairment

The result of the leave-one-out analysis shows that omitting study by Bartels et al. (2021) causes the overall prevalence of CRCI to decrease by 23%, while the overall prevalence of 12 studies was 26% (Fig. 4). The analysis did not show any evidence of small-study effects (Egger’s test: *p* = 0.9191).

## Discussion

This meta-analysis represents the first attempt to summarize the overall prevalence of cognitive-related cancer-related impairment (CRCI) among lung cancer survivors. The result indicates the prevalence of CRCI in lung cancer survivors was 26%, which is similar to breast cancer survivors (around 15–25%) [5].

Of 12 studies involving 1934 lung cancer survivors, 33.3% reported that CRCI was assessed by neuropsychological tests using the ICCTF criteria, 25% applied the MMSE, and 8.3% reported the application of the SPMSQ. In these neuropsychological tests, cognitive domains including verbal learning and memory, verbal fluency, expressive and receptive language, visuospatial memory, working memory, attention, executive function, processing speed and visual attention, mental flexibility, verbal and visual reasoning, and motor coordination were found to be applied in assessing cognitive function in lung cancer survivors. In line with the six key domains of cognitive function defined by the Diagnostic and Statistical Manual of Mental Disorders 5th edition (DSM-5) [39], most major domains were covered; however, the social cognition and emotions domain was not reported in the included studies. It is noted that the Cambridge Neuropsychological Test Automated Battery (CANTAB) was used in an included study partially (for measuring visuospatial memory only) [29]. In fact, the CANTAB test batteries include emotion and social cognition domain such as Emotion Bias Task (EBT) and Emotion Recognition Task (ERT) to measure distinct aspects of social cognition [40]. Future research may consider including emotion and social cognition domain to perform comprehensive neuropsychological tests for assessing cognitive function in lung cancer survivors.

As for the subjective assessments measuring perceived cognitive impairment for CRCI, three studies applied subjective assessments including CogPCI subscale and EORTC QLQ-C30 cognitive function subscale. Within CRCI measurement, the classical test theory is still dominant in terms of the development of most survivor-reported outcome measures [19]. Due to the inherent limitations of simpler assessments, there has been a growing trend towards the use of more complex psychometric instruments and methodologies for administering patient-reported outcomes [41]. Item response theory and computerized adaptive testing are sophisticated techniques for measuring patient outcomes in various fields of health care, but they have not been widely employed in CRCI assessment. Item response theory is important to the approach of the Patient-Reported Outcomes Measurement Information System (PROMIS) effort of the National Institutes of Health, and more publicly available open-source psychometric software for completing analyses [42]. Item response theory can assist decide which items in a survey are best for measuring degrees of perceived cognitive impairment in the evaluation of CRCI [19, 43].

Further, in the subgroup analysis, we pooled the subgroup prevalences according to region (Asia/Europe/North America) and types of assessment (objective/subjective). Studies conducted in Asia (15%) shows lower prevalence of CRCI than in Europe (33%) and North America (44%). Despite the fact that only two studies from North America were included in the analysis, they were still considered and weighted using the double arcsine transformation of proportions, allowing for meaningful comparison with other studies. It is noted that the prevalences of CRCI among lung cancer survivors are higher, which are therefore crucial for clinicians to be aware of. In addition, the prevalences of CRCI by objective measure using comprehensive evaluations, objective measure using screening tool alone, and subjective measures were 36%, 17%, and 22%, respectively. The possible reason might be the nature of using objective measure using screening tool alone and subjective assessments, as those items were somehow limited. As aforementioned, two studies adopted EORTC QLQ-C30 cognitive functioning subscale that only involved attention and memory domains. It might not be able to capture all functioning domains of cognition. In line with the recommendations by ICCTF [7], five studies adopted a battery of neuropsychological assessments to perform a comprehensive evaluation of CRCI. Therefore, this review summarized the neuropsychological assessments adopted to assess CRCI in lung cancer survivors as a reference for future research or healthcare professionals to consider.

The meta-regression analysis revealed that the study region was a source of heterogeneity in this meta-analysis. To further investigate the impact of outliers, a sensitivity analysis was conducted. It was found that by removing one study, the overall prevalence of CRCI decreased from 26 to 23%. This suggests that the overall prevalence of CRCI may be even higher than initially estimated, which highlights the need for further attention and research in this area. Future studies should focus on developing preventive strategies and interventions specifically for lung cancer survivors to address this issue.

### Limitations

This meta-analysis has unavoidable limitations. First, it is important to note that the accuracy of the overall findings in this meta-analysis may have been impacted by the limited number of studies available in certain subgroups. The dearth of studies in these subgroups can introduce potential biases and limitations to the analysis. The smaller sample size can affect the precision and generalizability of the results. Second, while the majority of instruments used in the studies included in the analysis were validated, variations in measurement methods and assessors still exist; in particular, one study that reviewed medical record did not report how CRCI was identified. Moreover, the included studies did not fully report the assessor training for the use of neuropsychological tests and cognitive assessment tools. The absence of detailed information regarding measurement methods and assessors introduces the possibility of low reliability and inconsistent accuracy in detecting CRCI. Lastly, relevant studies in other languages were overlooked; thus, the generalizability of the study result may be limited.

Nevertheless, this meta-analysis possesses several notable strengths. One strength lies in the meticulousness of the search strategies, utilization of validated appraisal tools, and incorporation of studies from diverse geographic regions. These factors greatly enhance the internal and external validity of the meta-analysis. It is also important to highlight that none of the studies included in this analysis was found to have a high risk of bias. In order to account for the anticipated variability in proportional meta-analyses, several sensitivity analyses were performed in this study. These analyses included predefined subgroup analysis and meta-regression, which were carried out to explore and understand the sources of the observed heterogeneity. Moreover, outlier detection and leave-one-out analysis were performed to assess the influence of individual studies on the overall findings. These analytical approaches strengthen the robustness of the results and provide a more comprehensive understanding of the data. Furthermore, the robustness and reliability of the evidence collected in this meta-analysis were evaluated through the implementation of outlier detection and leave-one-out analysis.

## Conclusion

In this meta-analysis, which included a total of 12 studies, it was found that the combined prevalence of CRCI among lung cancer survivors was estimated to be 26%. The analysis also highlighted the significant impact of study region on the observed heterogeneity. There is a need for evidence-based interventions and policies that focus on preventing and reducing the prevalence of CRCI. By fostering international understanding of CRCI among clinicians, valuable knowledge and access to established protocols can be obtained, facilitating the evaluation of preventive strategies for CRCI. Considering the limited number of studies focusing on cognitive performance as a primary outcome in cancer survivorship and the growing population of lung cancer survivors worldwide, this study contributes to the existing epidemiological evidence in the field of lung cancer research. Therefore, the findings of this meta-analysis can play a vital role in raising awareness among healthcare professionals and researchers in both clinical and research settings.

### Supplementary information

ESM 1 (DOCX 341 kb)

## References

[1] Siegel RL, Miller KD, Wagle NS, Jemal A (2023). Cancer statistics, 2023. CA Cancer J Clin.

[2] Howington JA, Blum MG, Chang AC, Balekian AA, Murthy SC (2013). Treatment of stage I and II non-small cell lung cancer: diagnosis and management of lung cancer, 3rd ed: American College of Chest Physicians evidence-based clinical practice guidelines. Chest.

[3] Barta JA, Powell CA, Wisnivesky JP (2019) Global epidemiology of lung cancer. Ann Glob Health 85(1):8. Published 2019 Jan 22. 10.5334/aogh.2419 10.5334/aogh.2419 PMC6724220 30741509

[4] Ho MH, Lin CC (2022). The cancer-related symptoms puzzle: piecing cancer-related cognitive impairment to cancer care research. Cancer Nurs.

[5] Lange M, Joly F, Vardy J, Ahles T, Dubois M, Tron L, Winocur G, De Ruiter MB, Castel H (2019). Cancer-related cognitive impairment: an update on state of the art, detection, and management strategies in cancer survivors. Ann Oncol.

[6] Binarelli G, Duivon M, Joly F, Ahmed-Lecheheb D, Lange M (2023) Cancer-related cognitive impairment: current perspectives on the management of cognitive changes following cancer treatment. Expert Rev Neurother 1-20 10.1080/14737175.2023.2187288 36951414

[7] Wefel JS, Vardy J, Ahles T, Schagen SB (2011). International Cognition and Cancer Task Force recommendations to harmonise studies of cognitive function in patients with cancer. Lancet Oncol.

[8] Janelsins MC, Kesler SR, Ahles TA, Morrow GR (2014). Prevalence, mechanisms, and management of cancer-related cognitive impairment. Int Rev Psychiatry.

[9] Fleming B, Edison P, Kenny L (2023). Cognitive impairment after cancer treatment: mechanisms, clinical characterization, and management. BMJ.

[10] Jassem J, Penrod J, Goren A, Gilloteau I, Penrod JR (2015). Caring for relatives with lung cancer in Europe: an evaluation of caregivers’ experience. Qual Life Res.

[11] Bai L, Yu E (2021). A narrative review of risk factors and interventions for cancer-related cognitive impairment. Ann Transl Med.

[12] Mackenzie L, Marshall K (2021). Effective non-pharmacological interventions for cancer related cognitive impairment in adults (excluding central nervous system or head and neck cancer): systematic review and meta-analysis. Eur J Phys Rehabil Med.

[13] Schaffrath N, Oberste M, Zimmer P (2017). Effects of exercise interventions and physical activity behavior on cancer-related cognitive impairments: an update. Curr Opin Support Palliat Care.

[14] Campbell KL, Zadravec K, Bland KA, Chesley E, Wolf F, Janelsins MC (2020). The effect of exercise on cancer-related cognitive impairment and applications for physical therapy: systematic review of randomized controlled trials. Physical Therapy.

[15] Shahid M, Kim J (2020) Exercise may affect metabolism in cancer-related cognitive impairment. Metabolites 10(9) 10.3390/metabo10090377 PMC7570125 32962184

[16] Zimmer P, Baumann FT, Oberste M, Wright P, Garthe A, Schenk A, Elter T, Galvao DA, Bloch W, Hübner ST, Wolf F (2016). Effects of exercise interventions and physical activity behavior on cancer related cognitive impairments: a systematic review. Biomed Res Int.

[17] Binarelli G, Joly F, Tron L, Lefevre Arbogast S, Lange M (2021) Management of cancer-related cognitive impairment: a systematic review of computerized cognitive stimulation and computerized physical activity. Cancers (Basel) 13(20) 10.3390/cancers13205161 PMC8534081 34680310

[18] Zeng Y, Dong J, Huang M, Zhang JE, Zhang X, Xie M, Wefel JS (2020). Nonpharmacological interventions for cancer-related cognitive impairment in adult cancer patients: a network meta-analysis. Int J Nurs Stud.

[19] Henneghan AM, Van Dyk K, Kaufmann T, Harrison R, Gibbons C, Heijnen C, Kesler SR (2021). Measuring self-reported cancer-related cognitive impairment: recommendations from the cancer neuroscience initiative working group. J Natl Cancer Inst.

[20] Page MJ, McKenzie JE, Bossuyt PM, Boutron I, Hoffmann TC, Mulrow CD, Shamseer L, Tetzlaff JM, Akl EA, Brennan SE, Chou R, Glanville J, Grimshaw JM (2021). The PRISMA 2020 statement: an updated guideline for reporting systematic reviews. Bmj.

[21] Stroup DF, Berlin JA, Morton SC, Olkin I, Williamson GD, Rennie D, Moher D, Becker BJ, Sipe TA, Thacker SB (2000). Meta-analysis of observational studies in epidemiology: a proposal for reporting. Meta-analysis Of Observational Studies in Epidemiology (MOOSE) group. Jama.

[22] Munn Z, Moola S, Lisy K, Riitano D, Tufanaru C (2015). Methodological guidance for systematic reviews of observational epidemiological studies reporting prevalence and cumulative incidence data. Int J Evid Based Healthc.

[23] Wells GA, Shea B, O’Connell D, Peterson J, Welch V, Losos M, Tugwell P (2000) The Newcastle-Ottawa Scale (NOS) for assessing the quality of nonrandomised studies in meta-analyses, Oxford)

[24] Herzog R, Álvarez-Pasquin MJ, Díaz C, Del Barrio JL, Estrada JM, Gil Á (2013). Are healthcare workers' intentions to vaccinate related to their knowledge, beliefs and attitudes? A systematic review. BMC Public Health.

[25] Nyaga VN, Arbyn M, Aerts M (2014). Metaprop: a Stata command to perform meta-analysis of binomial data. Arch Public Health.

[26] Migliavaca CB, Stein C, Colpani V, Barker TH, Ziegelmann PK, Munn Z, Falavigna M (2022). Meta-analysis of prevalence: I(2) statistic and how to deal with heterogeneity. Res Synth Methods.

[27] Grosshans DR, Meyers CA, Allen PK, Davenport SD, Komaki R (2008). Neurocognitive function in patients with small cell lung cancer: effect of prophylactic cranial irradiation. Cancer.

[28] Lu Y, Liu X (2023). Postoperative delirium and its influencing factors in elderly patients with lung cancer in the intensive care unit. J Thorac Dis.

[29] Ma Y, Liu N, Wang Y, Zhang A, Zhu Z, Zhang Z, Li Y, Jian G, Fu G, Dong M, Zheng G, Zhu P, Zhong G (2023). Cognitive adverse events in patients with lung cancer treated with checkpoint inhibitor monotherapy: a propensity score-matched analysis. EClinicalMedicine.

[30] Schulkes KJ, Souwer ET, Hamaker ME, Codrington H, Sar-van der Brugge S, Lammers JJ, Portielje JE, van Elden LJ, van den Bos F (2017). The effect of a geriatric assessment on treatment decisions for patients with lung cancer. Lung..

[31] Zeng H, Hendriks LEL, Witlox WJA, Groen HJM, Dingemans AC, Praag J, Belderbos J, Houben R, van der Noort V, De Ruysscher DKM (2023). Risk factors for cognitive impairment in radically treated stage III NSCLC: secondary findings of the NVALT-11 study. Radiother Oncol.

[32] Gal O, Dudnik E, Rotem O, Finkel I, Peretz I, Zer A, Mandel J, Amiel A, Siegal T, Bar J, Lobachov A, Yust S (2020) Tyrosine kinase inhibitors as a treatment of symptomatic CNS metastases in oncogene-driven NSCLC. J Oncol:1–8 10.1155/2020/1980891 PMC7486631 32963526

[33] Luo J, Liu R, Luo Y, Fang Q, Liu S, Yang Z, Miao J, Zhang L (2023). The high burden of symptoms associated with cognitive impairment in lung cancer patients: a latent class analysis. Asia Pac J Oncol Nurs.

[34] Shi HX, Du XJ, Wu F, Hu YJ, Mi WD (2020). Dexmedetomidine for early postoperative cognitive dysfunction after video-assisted thoracoscopic lobectomy in elderly male patients with lung cancer. Medicine (Baltimore).

[35] Takemura N, Ho MH, Cheung DST, Lin CC (2022). Factors associated with perceived cognitive impairment in patients with advanced lung cancer: a cross-sectional analysis. Support Care Cancer.

[36] Eggen AC, Richard NM, Bosma I, Jalving M, Leighl NB, Liu G, Mah K, Higazy R, Shultz DB, Reyners AKL, Rodin G, Edelstein K (2022). Factors associated with cognitive impairment and cognitive concerns in patients with metastatic non-small cell lung cancer. Neurooncol Pract.

[37] Bartels F, Wandrey MM, Aigner A, Strönisch T, Farmer K, Rentzsch K, Tessmer A, Grohé C, Finke C (2021). Association between neuronal autoantibodies and cognitive impairment in patients with lung cancer. JAMA Oncol.

[38] Soria-Comes T, Palomar-Abril V, Martín Ureste M, García Sánchez J, Marco Buades JE, Fernández Llavador MJ, López Gabaldón A, González Jurado M, Maestu Maiques IC (2020). Cognitive impairment is related to a reduced count of T-lymphocytes in older patients diagnosed with non-small cell lung cancer (NSCLC). Transl Cancer Res.

[39] Sachdev PS, Blacker D, Blazer DG, Ganguli M, Jeste DV, Paulsen JS, Petersen RC (2014). Classifying neurocognitive disorders: the DSM-5 approach. Nat Rev Neurol.

[40] Cognition C (2023). Emotion & social cognition.

[41] Cella D, Yount S, Rothrock N, Gershon R, Cook K, Reeve B, Ader D, Fries JF, Bruce B, Rose M (2007). The Patient-Reported Outcomes Measurement Information System (PROMIS): progress of an NIH Roadmap cooperative group during its first two years. Med Care.

[42] Cella D, Riley W, Stone A, Rothrock N, Reeve B, Yount S, Amtmann D, Bode R, Buysse D, Choi S, Cook K, Devellis R, DeWalt D (2010). The Patient-Reported Outcomes Measurement Information System (PROMIS) developed and tested its first wave of adult self-reported health outcome item banks: 2005-2008. J Clin Epidemiol.

[43] Lai JS, Butt Z, Zelko F, Cella D, Krull KR, Kieran MW, Goldman S (2011). Development of a parent-report cognitive function item bank using item response theory and exploration of its clinical utility in computerized adaptive testing. J Pediatr Psychol.

