# Supplementary material for: Prevalence and assessment tools of cancer-related cognitive impairment in lung cancer survivors: a systematic review and proportional meta-analysis
Source: Support Care Cancer. 2024 Mar 5;32(4):209. doi: 10.1007/s00520-024-08402-9 (PMC10914843; doi:10.1007/s00520-024-08402-9)
Supplement: Supplementary file 1 — (DOCX 341 kb) [file 520_2024_8402_MOESM1_ESM.docx]

**SUPPORTING INFORMATION**

**Table S1.** Newcastle-Ottawa Scale Scoring of Non-randomized trials in the Meta-analysis

| First author, year | Selection | Comparability | Outcome | Total Score | Decision |
| --- | --- | --- | --- | --- | --- |
| Bartels, 2021^a^ | ★★★★★ | ★★ | ★★★ | 10/10 | Included |
| Eggen, 2022^a^ | ★★★★☆ | ★★ | ★★★ | 9/10 | Included |
| Gal, 2020^b^ | ★★★☆ | ★★ | ★★☆ | 7/9 | Included |
| Grosshans, 2008^b^ | ★★★☆ | ★★ | ★★★ | 8/9 | Included |
| Lu, 2023^b^ | ★★★★ | ★★ | ★★★ | 9/9 | Included |
| Luo, 2022^a^ | ★★★★☆ | ★☆ | ★★☆ | 7/10 | Included |
| Ma, 2023^b^ | ★★★★ | ★★ | ★★★ | 9/9 | Included |
| Schulkes, 2017^b^ | ★★★★ | ★☆ | ★★☆ | 7/9 | Included |
| Shi, 2020^b^ | ★★★★ | ★★ | ★★☆ | 8/9 | Included |
| Soria-Comes, 2020^b^ | ★★★☆ | ★★ | ★★★ | 8/9 | Included |
| Takemura, 2022^a^ | ★★★★★ | ★☆ | ★★☆ | 8/10 | Included |
| Zeng, 2023^b^ | ★★★★ | ★★ | ★★★ | 9/9 | Included |

***Note.*** ^a^Cross-sectional: Scores are 0–5 stars for selection, 0–2 stars for comparability, and 0–3 stars for outcome. Studies scored below 4 of the total score were considered having unsatisfactory methodological quality; ^b^Cohort: Scores are 0–4 stars for selection, 0–2 stars for comparability, and 0–3 stars for outcome. Studies scored 0 or 1 star in selection domain, or 0 stars in comparability domain, or 0 or 1 stars in outcome/exposure domain were considered having poor methodological quality.

**Table S2** Combinations of search keywords

**1. PubMed**

| #1 | Cogniti* [Text] |
| --- | --- |
| #2 | Cognitive Dysfunction [Mesh] |
| #3 | Cancer-related Cognitive Impairment [Title/Abstract] |
| #4 | Lung Neoplasms [Mesh] |
| #5 | Lung Cancer [Title/Abstract] |
| #6 | #1 OR #2 OR #3 |
| #7 | #4 OR #5 |
| #8 | #6 AND #7 |

**2. Cochrane Library**

| #1 | Cognitive |
| --- | --- |
| #2 | Cancer-related Cognitive Impairment |
| #3 | Cognitive Dysfunction [Mesh] |
| #4 | Lung Cancer |
| #5 | Cancer Patients |
| #6 | Cancer Survivors |
| #7 | Lung Neoplasms [Mesh] |
| #8 | #1 OR #2 OR #3 |
| #9 | #4 OR #5 OR #6 OR #7 |
| #10 | #8 AND #9 |

**3. EMBASE (via Ovid)**

| #1 | 'Cogniti*':ti,ab,kw |
| --- | --- |
| #2 | 'Cognitive':ti,ab,kw |
| #3 | 'Cancer-related Cognitive Impairment':ti,ab,kw |
| #4 | 'Lung Cancer':ti,ab,kw |
| #5 | #1 OR #2 OR #3 |
| #6 | #4 AND #5 |

**4. CINAHL (via EbscoHost)**

| #1 | AB Cogniti* |
| --- | --- |
| #2 | MH Cognitive Dysfunction |
| #3 | AB Cancer-related Cognitive Impairment |
| #4 | AB Lung Cancer |
| #5 | MH Lung Neoplasms |
| #6 | #1 OR #2 OR #3 |
| #7 | #4 OR #5 |
| #8 | #6 AND #7 |

**5. China National Knowledge Infrastructure (CNKI)**

| #1 | TKA=认知 |
| --- | --- |
| #2 | TKA=肿瘤 |
| #3 | TKA=肺癌 |
| #4 | #2 OR #3 |
| #5 | #1 AND #4 |

**
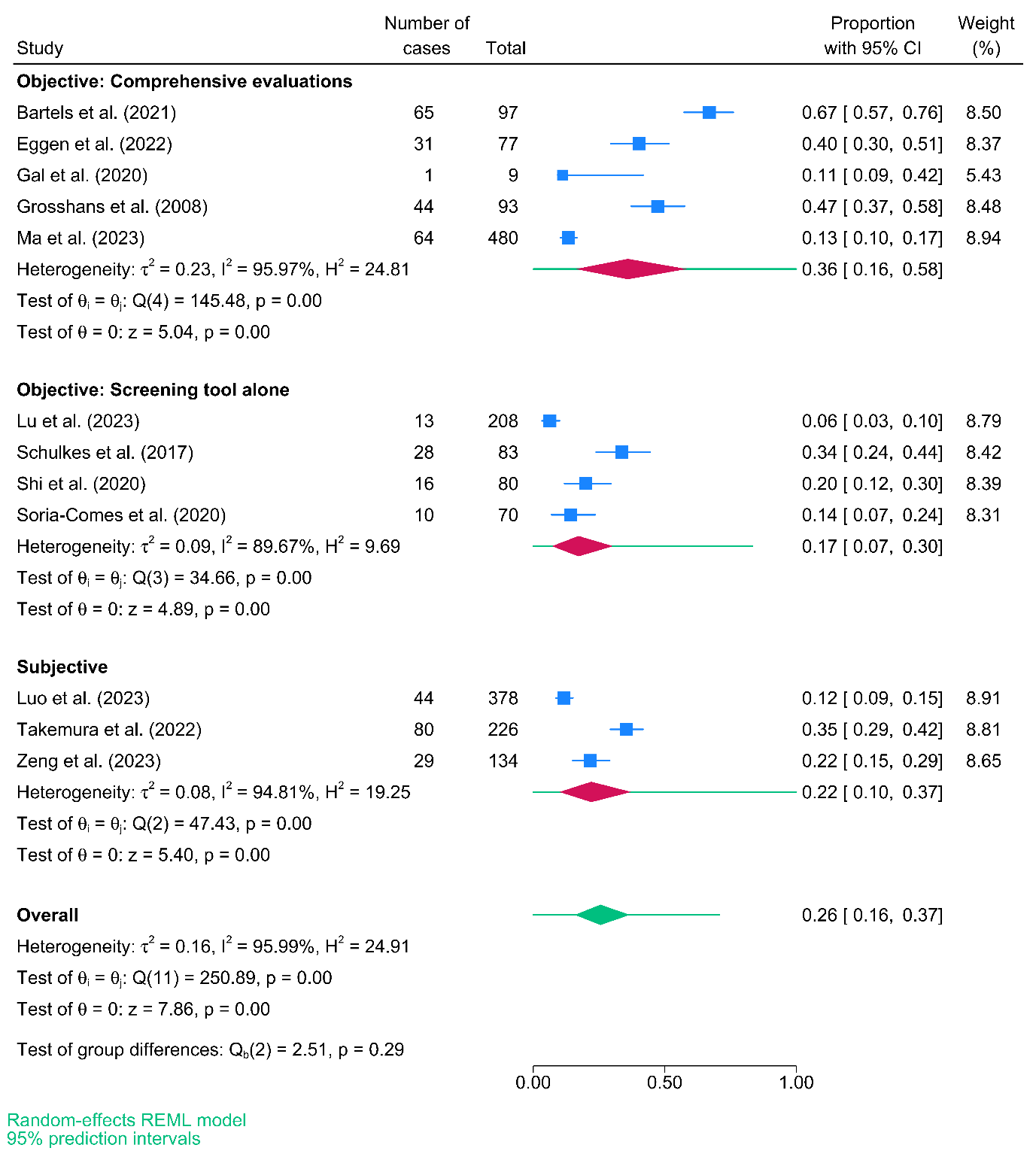
**

**Figure S1.** The forest plot of the overall pooled prevalence, subjective, and objective measures reported estimates of cancer-related cognitive impairment from a random-effects model.
